# A regression based phase I clinical trial for late-onset toxicities without clinician elicitation

**DOI:** 10.1016/j.conctc.2019.100327

**Published:** 2019-01-30

**Authors:** Andrew G. Chapple, Janusz J. Wojcik, Lee S. McDaniel

**Affiliations:** Louisiana State University Health Sciences Center, New Orleans, 2020 Gravier Street, New Orleans, LA, 70112, USA

**Keywords:** Phase I, Clinical trials, Dose-finding, Time-to-event, Isotonic regression

## Abstract

An extension of the isotonic regression based phase I clinical trial design is presented that incorporates partial follow-up times into estimation of the raw toxicity probabilities. This phase I clinical trial design, called the TITE-IR design, drastically decreases average trial duration by allowing patients to be treated immediately after being enrolled in a phase I clinical trial. The TITE-IR design does not require specification of a prior skeleton of toxicity probabilities like the continual reassessment method, has an additional trial parameter for controlling aggressiveness of dose escalation, and has an easily understood formula for estimating toxicity probabilities. An R statistical software package is described in detail in the appendix for simulating and implementing the design. A simulation study shows that the TITE-IR design outperforms the 3 + 3 design in terms of selecting the true maximum tolerated dose and results in shorter trial times, without a large loss in efficiency, compared to the isotonic regression design and Storer's up-and-down design D. These properties make the TITE-IR design a more appealing option to clinicians than the two most commonly used 3 + 3 designs and the isotonic regression design with larger follow-up windows for toxicity.

## 1 Introduction

Phase I trials in oncology aim to find a dose level among a set of doses that has a toxicity probability closest to a pre-defined target probability. This dose, called the maximum tolerated dose (MTD), is chosen to be the highest dose level with some acceptable degree of toxicity probability. There have been a myriad of complicated phase I trials proposed in the statistical literature over the past three decades, but they have not been widely adopted. Clinical trials are frequently still run using the 3 + 3 design, even though this design has shown major flaws in terms of picking the best dose for patients. Variations, e.g. Storer's Up-and-Down design D [1], of this design are easy for clinicians running the trial to understand as they have simple escalation and de-escalation rules based on toxicity responses of 3 or 6 patients.

O'Quigley et al. [2] and O'Quigley and Shen [3] established the first model based alternative to the 3 + 3 design with the Continual Reassessment Method (CRM), which required clinician specification of prior toxicity beliefs for each dose level. Cheung and Chappell [4] extended the CRM to incorporate partial patient follow-up information, which drastically reduced average trial durations compared to the CRM. This is evident especially in cases with long patient follow-up windows for toxicity. For example, some chemotherapy toxicities may manifest within a year, delaying drug development if full follow-up is required.

All three of these designs require some prior elicitation from clinicians, usually in the form of prior probabilities of toxicity for each dose by the desired follow-up time. While these designs give superior results to the traditional 3 + 3 design [5], the CRM designs have not been widely adopted. Hansen et al. [6] reviewed 151 phase I trials conducted during 2000–2010, of which 62% were 3 + 3 designs, 30% were accelerated titration designs and only 5% used the CRM. These figures demonstrate the unwillingness of clinicians to use more complicated designs like the CRM.

Leung and Wang [7] introduced a phase I trial based on isotonic regression that does not require specification of prior toxicity probabilities by clinicians. The design does not assume a functional relationship between the dose levels and the toxicity probabilities other than monotonicity (toxicity probability must not decrease with dose), showing that in certain scenarios this approach may outperform the CRM.

We propose a new design, TITE-IR, which extends the isotonic regression design by estimating toxicity probabilities as a function of follow-up time, the proportion of toxicities seen at each dose, and an additional user specified parameter that controls the aggressiveness of dose escalation. A simulation study shows that this design outperforms the 3 + 3 and Up-and-Down designs, and results in shorter average trial duration compared to competitors. Our method is implemented in the R package titeIR, which is easy for clinicians to use, with a tutorial seen in the appendix [8].

## 2 Model and trial conduct

We consider settings where we have *J* doses to consider in a phase I trial with raw dose levels (*d*_1_*,…,d*_*J*_) and we will enroll a maximum of *N*_max_ patients in the trial. We wish to find the dose that has a toxicity probability less than but closest to a target toxicity probability *π*^∗^ (1/3, for example) by some time *τ*. In the traditional isotonic phase I trial of Leung and Wang [7]; they estimate probabilities of toxicity as ${\hat{q}}_{1},\ldots ,{\hat{q}}_{J}$ using the proportion of toxicities for patients treated at each dose. They use isotonic regression to impose a monotonicity constraint, ensuring that the final estimated toxicity probabilities, ${\hat{p}}_{1},\ldots ,{\hat{p}}_{J}$, increase with dose level.

This strategy does not result in appreciable delay if *τ*, the necessary follow-up time, is small. However, if *τ* is large relative to the time between patient arrival, patients may have to wait to be assigned to doses while a previous cohort is followed. Let *n*_*j*_ denote the number of patients who have been treated at dose *j* and denote *Y*_*j*_ as a random variable representing the total number of toxicities for dose *j* (with observed *y*_*j*_). If *t*_*i*_ ≤ *τ* is the follow-up time for patient *i* (i.e. we stop following them for toxicity after *τ*), we propose estimating ${\hat{q}}_{1},\ldots ,{\hat{q}}_{J}$ as:

$${\hat{q}}_{j}=\frac{Y_{j}+\sum_{i:Y_{ij}=0}\left(\frac{\tau -t_{j}}{\tau }\right)\left(\pi^{*}+F^{*}\right)}{n_{j}},$$

where *Y*_*ij*_ is the binary indicator of toxicity for patient *i*, treated with dose *d*_*j*_. We offset the traditional estimate *Y*_*j*_*/n*_*j*_ by (*π*^∗^ + *F*^∗^)(*τ* − *t*_*j*_)/(*τn*_*j*_) here because we assume prior to the trial that each dose is equally likely to be the MTD and that as a patient is followed closer to *τ*, the probability of toxicity becomes less likely. Specifically, we assume that the time until a toxicity is uniformly distributed from 0 to *τ*. *F*^∗^ *>* 0 is used to make the trial more conservative in terms of dose assignment, with larger values making dose escalation more difficult. We suggest a default value of *F*^∗^ = 0.05, but *F*^∗^ can be calibrated to make the design safer, while sacrificing some accuracy. To calibrate *F*^∗^ we simulate the design under one scenario where the lowest dose is acceptable and all others are too toxic based on *π*^∗^. We also simulate under one scenario where the largest or second largest dose is the closest to *π*^∗^ and calibrate *F*^∗^ such that the proportion of patients treated above the MTD in the toxic scenario is reasonable without sacrificing optimal dose selection probability in the second described scenario. We then use isotonic regression on the estimates ${\hat{q}}_{1},\ldots ,{\hat{q}}_{J}$ to estimate the probability of toxicity by *τ* months for each dose as ${\hat{p}}_{1},\ldots ,{\hat{p}}_{J}$, which imposes the monotonicity constraint. Specifically, if the estimate of DLT probability at a dose is lower than the estimate of DLT probability at the next lower dose (a violation of monotonicity), then the information from the two doses is pooled together and a new estimate is calculated assuming the two doses have the same toxicity probability [9,10].

During the trial, we enroll the next patient cohort to the dose *d*^opt^ that has the closest toxicity probability to *π*^∗^, without being greater than *π*^∗^. If there are ties, the optimal dose is taken to be the highest dose satisfying these conditions. Formally, this is

$$d^{opt}=.\begin{array}{c} d_{j}:\mathrm{maxargmin}\left|\pi^{*}-{\overset{⌢}{p}}_{j}\right|\\ j:{\overset{⌢}{p}}_{j}\leq \pi^{*} \end{array}.$$

After *N*_max_ patients are enrolled in the trial, we follow them until toxicity or *τ* and declare the MTD to be *d*^opt^. We start every trial at the lowest dose and do not allow escalation until at least three patients have been fully followed at each dose to ensure a safer trial. We also do not skip untried doses when escalating and do not allow for escalation if the most recently assigned patient has had a toxicity.

## 3 Simulation study

We perform a simulation study to demonstrate the proposed method's performance compared to the traditional 3 + 3 design, the Up-and-Down design D of Storer [1] and the phase I isotonic regression design that does not incorporate partial follow-up information. For the purpose of this simulation, we consider 6 doses of a new agent given orally at raw dose levels (*d*_1_*,d*_2_*,d*_3_*,d*_4_*,d*_5_*,d*_6_) = (5,10,15,20,30,40) milligrams. We define the MTD as the dose level that has a toxicity probability by *τ* = 6 months closest to but less than our target of *π*^∗^ = 1/3. We enroll up to *N*_max_ = 24 patients and assume that we accrue 2 patients per month, generated from a Poisson process. We examine 10 different scenarios, with the first 8 coming from Leung and Wang [7]; and the last 2 scenarios designed to be structured differently from the first 8. The true toxicity probabilities of these 10 scenarios for each dose are shown in Table 1. We generate toxicity times for patients that experienced toxicity uniformly from 0 to *τ*.

**Table 1:** Simulation Study: True toxicity probabilities at 6 months for each dose considered in the trial.

Scenario	Dose 1	Dose 2	Dose 3	Dose 4	Dose 5	Dose 6
1	0.05	0.10	0.20	0.30	0.50	0.70
2	0.09	0.16	0.27	0.38	0.57	0.75
3	0.30	0.40	0.52	0.61	0.76	0.87
4	0.00	0.00	0.04	0.09	0.25	0.49
5	0.20	0.90	0.90	0.90	0.90	0.90
6	0.10	0.20	0.90	0.90	0.90	0.90
7	0.30	0.30	0.50	0.50	0.50	0.50
8	0.00	0.00	0.03	0.05	0.11	0.33
9	0.12	0.18	0.22	0.25	0.33	0.50
10	0.10	0.10	0.20	0.20	0.40	0.40

We enroll cohorts of size 3 for the 3 + 3 design, the Up-and-Down design, and the usual isotonic regression phase I designs. We compare our method to two isotonic regression phase I trials. One approach, called IR-B, does not allow for early stopping of the trial to declare an MTD. The IR-A design stops and declares an MTD if the same dose has been given to 3 consecutive cohorts and is indicated for the next cohort. This approach allows slightly faster trials while sacrificing some accuracy in choosing the MTD. For our proposed design we enroll patients as they are accrued in the trial, significantly reducing overall duration. We do not allow the TITE-IR design to stop early because the design results in much faster trials than its competitors. *F*^∗^ was calibrated under scenario 5, where doses 2–6 are too toxic such that the % of patients treated above the MTD was about the same as the 3 + 3 and UD designs without compromising the optimal dose selection probability in a scenario where the true MTD was high, such as scenario 8. This calibration resulted in the suggested default of 0.05.

We stop the 3 + 3 trials and declare an MTD based on the traditional rules. Formally, after enrolling a cohort of 3 patients at dose *d*_*j*_, if we see *>* 1 toxicity, we declare dose *d*_*j*_−1 the MTD (if *j* = 1, we declare dose 1 to be the MTD). If we see 0 toxicities, we escalate doses and assign the next patient to dose *d*_*j*+1_. If 1 toxicity is observed, then an expansion cohort is enrolled at dose *d*_*j*_ and if 0 toxicities are observed for the last cohort treated, we escalate to dose *d*_*j*+1_, otherwise we declare *d*_*j*_−1 the MTD. After *N*_max_ patients are enrolled in the trial, if we have yet to determine an MTD we may need an additional expansion cohort. If 0 toxicities are observed at dose *d*_*j*_ after *N*_max_ patients have been enrolled, we declare *d*_*j*_ the MTD. If 1 toxicity is observed at dose *d*_*j*_, we add one additional expansion cohort (making the trial sample size *N*_max_ + 3) and declare *d*_*j*_ the MTD with no toxicities, otherwise *d*_*j*_−1 is declared the MTD.

**Fig. 1:**
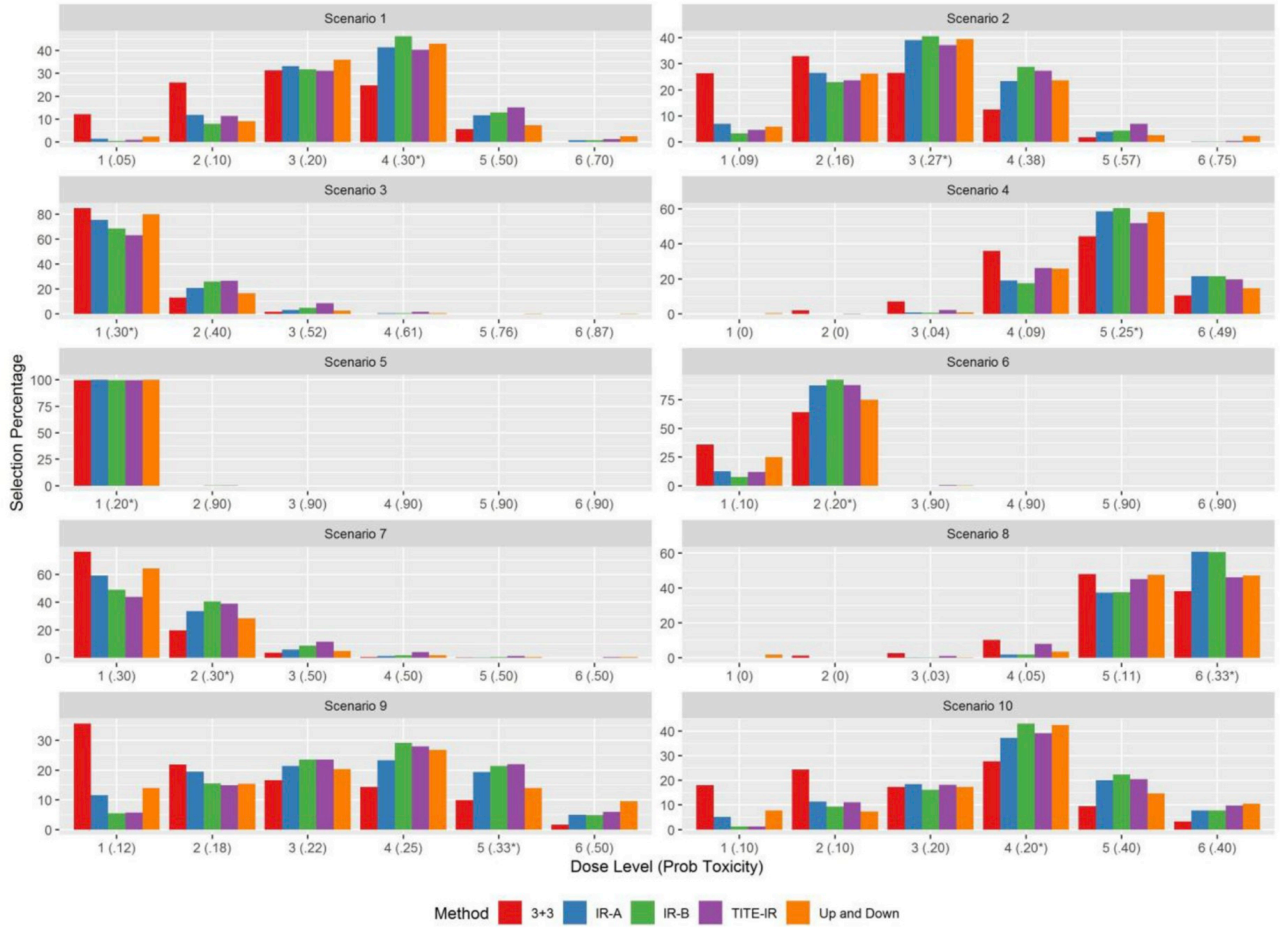
Simulation Study: Dose selection probabilities in each scenario. Prob Toxicity denotes the true toxicity probability for each dose and * denotes the dose toxicity probability closest to the target of 1/3 without exceeding 1/3.

For the Up-and-Down design D of Storer [1]; we enroll cohort sizes of 3 and if we see no toxicities at *d*_*j*_, we escalate to *d*_*j*+1_. If we see one toxicity, we treat the next cohort at *d*_*j*_ and if we see *>* 1 patient toxicities for dose *d*_*j*_, we de-escalate (unless *j* = 1). We continue the trial until *N*_max_ patients are enrolled in the trial. Storer [1] suggests fitting a simple logistic regression of the doses assigned to patients on their toxicity status to choose the MTD, but notes that often with small sample sizes the monotonicity constraint is not upheld. We declare the MTD to be the dose chosen based on the logistic regression on the final trial data. We compare the operating characteristics (OCs) of our proposed design to the aforementioned competitors by simulating 10,000 trials for each scenario and looking at the proportion of times the true MTD was chosen, the average sample size of each trial, the average number of toxicities, the average number of patients treated above and below the MTD, and the average trial time. Table 2, Table 3 display the operating characteristics of the 5 phase I trial designs and the dose selection % for each scenario are shown graphically in Fig. 1.

**Table 2:** Simulation Study: PCD = probability of selecting the correct dose. *Ntox* = average number of toxicities. *N* = average sample size. *Dur* is the average trial duration in years. *MTD* ↓, *MTD* and *MTD* ↑ denotes the average number of patients treated below, at, and above the maximum tolerated dose, respectively.

Scenario	Design	PCD	*Ntox*	*N*	*Dur*	*MTD* ↓	*MTD* =	*MTD* ↑
1	3 + 3	24.8	2.9	14.9	2.7	76.6	15.3	8.1
	UD	43.0	5.1	24.0	4.1	64.7	25.2	10.1
	IR-A	41.4	4.9	22.5	3.6	65.5	23.7	10.8
	IR-B	46.3	5.3	24.0	4.1	63.4	25.5	11.1
	TITE-IR	40.3	5.1	24.0	1.5	68.7	18.5	12.8
2	3 + 3	26.6	2.8	12.9	2.3	63.8	22.5	13.7
	UD	39.4	5.7	24.0	4.1	46.4	31.5	22.1
	IR-A	39.0	5.1	21.6	3.4	49.5	29.5	20.9
	IR-B	40.6	5.8	24.0	4.1	45.8	31.2	23.0
	TITE-IR	37.1	5.8	24.0	1.5	51.1	25.4	23.5
3	3 + 3	85.1	2.5	7.1	1.3	0	72.0	28.0
	UD	80.1	8.6	24.0	4.1	0	56.0	44.0
	IR-A	75.7	5.9	16.5	2.4	0	63.3	36.7
	IR-B	68.7	8.5	24.0	4.1	0	57.9	42.1
	TITE-IR	63.3	9.0	24.0	1.5	0	52.6	47.4
4	3 + 3	44.4	2.7	19.3	3.4	67.6	19.4	13.0
	UD	58.3	3.7	24.0	4.1	59.8	27.1	13.1
	IR-A	58.5	3.93	23.9	4.0	58.1	26.7	15.3
	IR-B	60.5	3.93	24.0	4.1	57.7	27.0	15.3
	TITE-IR	51.8	2.95	24.0	1.5	71.4	17.5	11.1
5	3 + 3	99.9	2.8	6.4	1.2	0	67.6	32.4
	UD	100	10.1	24.0	4.0	0	68.3	31.7
	IR-A	100	5.8	18.5	2.6	0	84.3	15.7
	IR-B	99.9	7.2	24.0	4.0	0	85.5	14.5
	TITE-IR	99.9	10.1	24.0	1.5	0	68.4	31.6

**Table 3:** Simulation Study: PCD = probability of selecting the correct dose. *Ntox* = average number of toxicities. *N* = average sample size. *Dur* is the average trial duration in years. *MTD* ↓, *MTD* and *MTD* ↑ denotes the average number of patients treated below, at, and above the maximum tolerated dose, respectively.

Scenario	Design	PCD	*Ntox*	*N*	*Dur*	*MTD* ↓	*MTD* =	*MTD* ↑
6	3 + 3	64.1	2.9	9.4	1.7	43.0	37.7	19.3
	UD	74.9	8.2	24.0	4.0	23.2	53.0	23.8
	IR-A	87.4	5.8	21.3	3.2	23.2	64.3	12.5
	IR-B	92.2	6.6	24.0	4.0	20.0	66.6	13.5
	TITE-IR	87.7	8.6	24.0	1.5	25.8	47.6	26.7
7	3 + 3	19.7	2.6	7.7	1.4	69.7	21.3	9.0
	UD	28.3	8.1	24.0	4.1	48.2	34.4	17.4
	IR-A	33.4	5.6	17.0	2.5	59.0	27.9	13.1
	IR-B	40.3	8.0	24.0	4.1	53.7	30.7	15.6
	TITE-IR	38.9	8.3	24.0	1.5	48.3	28.6	23.1
8	3 + 3	38.2	2.0	20.2	3.6	70.2	19.8	0
	UD	47.1	2.8	24.0	4.1	75.5	24.5	0
	IR-A	60.7	2.9	24.0	4.1	72.6	27.4	0
	IR-B	60.6	3.0	24.0	4.1	72.8	27.2	0
	TITE-IR	46.0	1.9	24.0	1.5	85.6	14.4	0
9	3 + 3	9.9	2.8	13.6	2.4	91.7	5.7	2.6
	UD	14.0	5.0	24.0	4.1	89.7	8.2	2.1
	IR-A	19.3	4.5	21.5	3.5	89.7	8.0	2.3
	IR-B	21.4	5.0	24.0	4.1	89.0	8.6	2.3
	TITE-IR	22.0	5.0	24.0	1.5	88.6	7.8	3.6
10	3 + 3	27.7	2.8	15.2	2.7	75.3	12.7	12.0
	UD	42.4	4.6	24.0	4.1	63.2	21.6	15.2
	IR-A	37.2	4.3	22.5	3.7	65.6	18.7	15.7
	IR-B	43.0	4.7	24.0	4.1	63.7	20.2	16.1
	TITE-IR	39.2	4.4	24.0	1.5	68.6	16.9	14.5

Table 2 shows that different designs perform best in different scenarios in terms of optimal dose selection probability. The IR-A design performs best in scenarios 5 (tie) and 9, and the IR-B design performs best in scenarios 1, 2, 4, 6, 7 and 10. The 3 + 3 design has the worst optimal dose selection probability in all but scenario 3. The TITE-IR design has the worst optimal dose selection probability in scenario 3 and the best in scenario 9. The TITE-IR design outperformed the 3 + 3 design in terms of the probability of selecting the true MTD by an average of 8.5% across the 10 scenarios considered. The design did suffer compared to the two isotonic regression designs, losing by an average of 2.6% and 4.7% to the IR-A and IR-B designs, respectively. The TITE-IR design performed as well on average as the up-and-down design D. This slight loss in efficiency of optimal dose selection compared to the two isotonic regression designs is warranted based on the drastic decrease in average trial duration. The TITE-IR design was completed in 2.3 fewer years than the IR-B design, which does not allow early stopping, and 1.8 fewer years compared to the IR-A design, which allows early stopping for declaring the MTD. The TITE-IR decreased the average trial length by 2.3 and 0.8 years, compared to the up-and-down design and 3 + 3 design, respectively. The average trial duration for the TITE-IR design is the same for each scenario because it only depends on the accrual rates, treating patients as soon as they enroll in the trial. Since the average selection probability of the MTD for each method is between 44 and 58%, using a design that decreases the trial duration is warranted for increased rates of drug development, which is achieved by the TITE-IR design.

The optimal dose selection probabilities are shown graphically in Fig. 1 along with the true toxicity probabilities for each dose along the x-axis. The toxicity probability that is closest to the target of 1/3, but below, has an asterisk to denote it as the MTD. The up-and down, IR-A, IR-B, and TITE-IR designs choose the MTD or one of the adjacent doses with the highest probability. The TITE-IR design picks doses higher than the MTD with a slightly higher probability in scenarios 1, 2, 3, and 7 than the opposing designs, but this difference is small. The TITE-IR design treated 1.5–5% more patients above the MTD than the two isotonic regression designs and the Up-and-Down design. The 3 + 3 design treated the smallest percentage of patients above the MTD due to its aggressive early stopping rule. The slight increase in treating patients above the MTD in the TITE-IR design results in more patient toxicities than the IR-B design, and toxicity rates that are similar to the Up-and- Down design. The TITE-IR design had the highest above MTD patient assignment rate in 6 scenarios, but was only higher than the respective rate of the next highest design by an average of 2.98% in these scenarios. The above MTD patient assignment rate was the highest in scenarios 3 and 7 where the lowest dose had a true toxicity probability of .3, suggesting that without having dose-toxicity information below the MTD there is an increased risk of treating patients above the MTD. This problem could be mitigated by designing trials that have a starting dose with an empirical toxicity probability below *π*^∗^ based on previous published studies.

Early stopping mitigates the overall toxicities in the IR-A design, while the percentage of patients treated above the MTD is similar to IR-B. Aggressiveness in dose escalation for TITE-IR is due to assignment of patients based on partial follow-up information, which could be mitigated by increasing *F*^∗^, but would sacrifice optimal dose selection probability. Depending on the severity of the patient toxicity expected, this trade-off may be warranted and can be calibrated using the function isotitesim from the R statistical software package titeIR, available on CRAN. It could be argued that although toxicities are increased, patients are not waiting to receive possibly life saving chemotherapy or other treatments. In our simulation study, patients could expect to wait between 1 and 5 months to receive treatment in the trial for the designs that treat cohort sizes of 3. The TITE-IR design eliminates this problem. We also performed a sensitivity analysis of the TITE-IR design to accrual rates of 1 and 3 patients per month as well as early and late-onset toxicities. These results are described in appendix Sections B and appendix Sectioncs C and Tables B1 and C1. In general, decreasing the accrual rate improves the operating characteristics due to more complete data and late onset toxicities increase the number of average observed toxicities by about 0.75. Since the other 4 designs do not make decisions without a fully evaluated patient cohort, the simulation results for these competitors do not change for different accrual rates or time to event distributions.

## 4 Discussion

We established a new phase I clinical trial design that extends the isotonic regression trial described by Leung and Wang [7]. The design, called TITE-IR, uses partial follow-up information of patients to estimate toxicity probabilities at each dose. The TITE-IR design treats patients as they are enrolled in the trial, which is critical for testing the safety of new therapies with delayed toxicities. Even with shorter toxicity windows, the TITE-IR design can give patients in the trial treatments immediately which could make a substantial difference in patient outcomes and recruitment. The TITE-IR design results in significant decreases in trial duration and out-performs the 3 + 3 design in simulations in terms of selecting the true MTD. Our simulation study showed the TITE-IR design had a slight increase in aggressiveness and decrease in optimal dose selection probability compared to the alternative isotonic regression, but this trade-off is warranted due to the decreased trial duration and the ability to treat patients as they arrive.

This design is easy to understand by clinicians as the probability estimates can be determined through a relatively simple formula, rather than via maximum likelihood or Bayesian methods, and a package has been developed, titeIR, for simulating and running the trial. This package is easy to use for clinicians and is described in detail in the appendix, along with examples. While designs that incorporate prior clinician opinions like the TITE-CRM or the Bayesian optimal interval design have excellent dose selection properties, clinicians have been uncomfortable making prior assessments of each dose levels toxicity probability. Furthermore, these designs are not well understood by clinicians, even though their superiority to the commonly used 3 + 3 and up-and-down designs are well documented. The TITE-IR design does not perform as well as these alternative approaches, but can serve as a stepping stone with equal or better operating characteristics than the commonly used phase I trial designs.

## Figures and Tables

**Table B1:** Simulation Study: PCD = probability of selecting the correct dose. *Ntox* = average number of toxicities. *N* = average sample size. *Dur* is the average trial duration in years. *MTD* ↓, *MTD* and *MTD* ↑ denotes the average number of patients treated at below, at, and above the maximum tolerated dose, respectively.

Scenario	Accrual	PCD	*Ntox*	*N*	*Dur*	*MTD* ↓	*MTD* =	*MTD* ↑
1	1/month	41.5	5.8	24	2.5	60.2	22.6	17.2
	2/month	40.3	5.1	24	1.5	68.7	18.5	12.8
	3/month	37.3	4.1	24	1.2	78.3	14.9	6.8
2	1/month	36.3	6.4	24	2.5	43.3	27.1	29.6
	2/month	37.1	5.8	24	1.5	51.1	25.4	23.5
	3/month	37.1	5.0	24	1.2	61.2	22.5	16.3
3	1/month	64.6	9.1	24	2.5	0	50.7	49.3
	2/month	63.3	9.0	24	1.5	0	52.6	47.4
	3/month	64.9	8.8	24	1.2	0	57.5	42.5
4	1/month	56.2	4.0	24	2.5	59.8	22.2	18.0
	2/month	51.8	3.0	24	1.5	71.4	17.5	11.1
	3/month	46.4	1.8	24	1.2	84.6	11.8	3.6
5	1/month	99.9	8.8	24	2.5	0	76.2	23.8
	2/month	99.9	10.1	24	1.5	0	68.4	31.6
	3/month	99.7	10.5	24	1.2	0	66.3	33.7
6	1/month	88.5	8.0	24	2.5	20.9	57.0	22.1
	2/month	87.7	8.6	24	1.5	25.8	47.6	26.7
	3/month	86.1	8.2	24	1.1	32.3	42.7	25.0
7	1/month	40.4	8.4	24	2.5	44.9	30.2	24.9
	2/month	38.9	8.3	24	1.5	48.3	28.6	23.1
	3/month	38.4	8.1	24	1.2	54.4	27.3	18.3
8	1/month	54.7	2.8	24	2.5	74.4	25.6	0
	2/month	46.0	1.9	24	1.5	85.6	14.4	0
	3/month	20.7	1.0	24	1.2	95.7	4.3	0
9	1/month	27.3	5.4	24	2.5	82.7	11.2	6.1
	2/month	22.0	5.0	24	1.5	88.6	7.8	3.6
	3/month	15.1	4.5	24	1.2	94.2	4.8	1.1
10	1/month	39.6	4.9	24	2.5	59.1	19.4	21.5
	2/month	39.2	4.4	24	1.5	68.6	16.9	14.5
	3/month	38.1	3.8	24	1.2	78.7	13.8	7.5

**Table C1:** Simulation Study: PCD = probability of selecting the correct dose for an accrual rate of 2 per month and different failure time distributions. *Ntox* = average number of toxicities. *N* = average sample size. *Dur* is the average trial duration in years. *MTD* ↓, *MTD* and *MTD* ↑ denotes the average number of patients treated at below, at, and above the maximum tolerated dose, respectively.

Scenario	Distribution	PCD	*Ntox*	*N*	*Dur*	*MTD* ↓	*MTD* =	*MTD* ↑
1	Weibull(1/2)	35.5	4.2	24	1.5	78.2	15.7	6.0
	Uniform	40.3	5.1	24	1.5	68.7	18.5	12.8
	Weibull(2)	41.5	6.0	24	1.5	59.7	19.8	20.5
2	Weibull(1/2)	38.2	4.9	24	1.5	62.4	24.5	13.1
	Uniform	37.1	5.8	24	1.5	51.1	25.4	23.5
	Weibull(2)	34.4	6.9	24	1.5	42.4	23.4	34.2
3	Weibull(1/2)	70.1	8.2	24	1.5	0	69.4	30.6
	Uniform	63.3	9.0	24	1.5	0	52.6	47.4
	Weibull(2)	59.8	9.9	24	1.5	0	41.0	59.0
4	Weibull(1/2)	49.6	2.5	24	1.5	76.3	16.8	7.0
	Uniform	51.8	3.0	24	1.5	71.4	17.5	11.1
	Weibull(2)	54.5	3.3	24	1.5	67.8	17.4	14.8
5	Weibull(1/2)	99.6	7.0	24	1.5	0	86.7	13.3
	Uniform	99.9	10.1	24	1.5	0	68.4	31.6
	Weibull(2)	99.8	11.3	24	1.5	0	61.3	38.7
6	Weibull(1/2)	84.3	5.9	24	1.5	33.2	55.7	11.2
	Uniform	87.7	8.6	24	1.5	25.8	47.6	26.7
	Weibull(2)	88.1	9.9	24	1.5	21.4	45.4	33.3
7	Weibull(1/2)	35.8	7.7	24	1.5	63.6	25.5	10.9
	Uniform	38.9	8.3	24	1.5	48.3	28.6	23.1
	Weibull(2)	39.0	8.9	24	1.5	36.7	28.3	35.0
8	Weibull(1/2)	40.3	1.7	24	1.5	88.6	11.4	0
	Uniform	46.0	1.9	24	1.5	85.6	14.4	0
	Weibull(2)	49.2	2.1	24	1.5	82.9	17.1	0
9	Weibull(1/2)	13.5	4.5	24	1.5	94.4	4.4	1.2
	Uniform	22.0	5.0	24	1.5	88.6	7.8	3.6
	Weibull(2)	28.7	5.5	24	1.5	81.1	11.6	7.3
10	Weibull(1/2)	35.7	3.9	24	1.5	78.0	14.3	7.7
	Uniform	39.2	4.4	24	1.5	68.6	16.9	14.5
	Weibull(2)	38.1	4.9	24	1.5	59.8	17.8	22.4

## Appendix A Use of the titeIR package

The titeIR package is available on CRAN and provides the necessary infrastructure for the design of phase 1 trials. To get started, an R session must be open (available for free download at https://www.R-project.org/). To install and load the package, run the following code:

install.packages("titeIR")

library(titeIR)

### Simulation

The isotitesim function can be used to simulate trial results with various different parameter settings and assumed toxicity probabilities at each dose. The isotitesim function has nine arguments. They are

• PI is a vector of true toxicity probabilities.
• target is the target toxicity probability for the MTD.
• n is the number of patients to enroll.
• nsim is the number of simulation replicates.
• obswin is the window of time over which the toxicity is observed.
• rate is the accrual rate, specifically the number of arrivals per observation window. For example, if obswin = 4 and rate = 2, we expect 2 patients to arrive in 4 time units.
• safety is *F*^∗^, the safety factor. The default value is 0.05.
• accrual is the accrual distribution. Acceptable values are "poisson" for a Poisson process or "fixed" for non-random arrival. Default is "poisson".
• restrict applies restrictions to escalation, ensuring that the design does not escalate if the most recent observation is a toxicity.

The TITE-IR design result for Scenario 1 in Table 2 can be reproduced by setting the seed:

set.seed(2389239)

scen1 = isotitesim(c(0.05, 0.10, 0.20, 0.30, 0.50, 0.70), target = 1/3, n = 24, nsims = 10000, obswin = 6, rate = 12)

scen1

which produces the result

Pr(DLT) 0.05 0.10 0.20 0.30 0.50 0.70

Selected 0.01 0.11 0.31 0.40 0.15 0.01

Nexpt 4.76 5.62 6.11 4.45 2.27 0.79

Ntox 0.23 0.57 1.24 1.33 1.13 0.57

The result shows that out of the 10,000 simulations (specified by the argument nsim = 10000), the dose with toxicity probability 0.2 was selected 31% of the time, and the dose with toxicity probability 0.3 was selected 40% of the time. The row Nexpt shows the average number of patients assigned to each dose during the trial. The final row, Ntox shows the average number of toxicities at each dose. A summary function provides a different view of the results:

summary(scen1)

which prints the result

Probability of Correct Dose: 0.4027

Average Number of Toxicities per trial: 5.0699

Duration: 17.97

Percent Treated below MTD: 68.7

Percent Treated at MTD: 18.5

Percent Treated above MTD: 12.8

### Dose Assignment

The isotitedose function provides, based on current data, the next dose recommended by the TITE-IR design. The isotitedose function has seven arguments. They are

• followup is a vector of followup times for the previously assigned patients.
• DLT is a vector of the same length as followup with 0 corresponding to no observed DLT and 1 corresponding to an observed DLT.
• assignment a vector of dose assignments which is the same length as followup. Doses should be labeled as consecutive integers starting from 1 corresponding to increasing dose levels.
• doses an integer corresponding to the number of doses.
• obswin is the window of time over which the toxicity is observed.
• target is the target toxicity probability for the MTD.
• safety is *F*^∗^, the safety factor. The default value is 0.05.

An example of usage is

isotitedose(followup = c(2, 1, 0.5), DLT = c(0, 0, 0),

assignment = c(1, 1, 1), obswin = 6, doses = 6)

which gives the recommended dose as 2, indicating an escalation. At this point in the trial, we have 3 patients with followup of 2, 1, and 1/2 time units. No DLTs have been observed, and all patients have been assigned to the first dose. The observation window is 6 time units, and there are 6 doses in total. The default values for target toxicity (1/3) and *F*^∗^ (0.05) are used.

## Appendix B Simulations with different accrual rates

In order to investigate the effect of accrual rate on performance of the proposed design, we varied the number of patients expected to arrive in each month. The simulations in Section 3 are based on an expected accrual rate of 2 patients per month, following a Poisson process, with an observation window of 6 months, leading to an expected 12 patients per observation window. In Table B1, we use the same simulation scenarios as Section 3, but with accrual rates of 1 patient per month and 3 patients per month.

From Table B1, we see that slower accrual typically leads to better performance. This is, of course, fully expected since assignment is based on more complete information. This is particularly true in scenarios such as scenario 8, in which the treatment is relatively safe. In this scenario, the built-in caution of TITE-IR is more effectively overcome by the more complete follow-up information.

## Appendix C Late and early onset toxicities

The simulations provided in Section 3 assumed a uniform distribution for the time to toxicity. We now repeat the simulations from with different failure time distributions. Table C1 includes the simulations assuming the uniform distribution along with two different Weibull distributions with hazard function given by

$$h\left(t\right)=\frac{\kappa }{\lambda }{\left(\frac{t}{\lambda }\right)}^{\kappa -1}$$

where *λ* is the scale parameter and *κ* is the shape parameter. The Weibull(1/2) has a shape parameter of 1/2 leading to a decreasing failure rate, while the scale parameter *λ* is chosen for each dose to achieve the appropriate toxicity probability. The Weibull(2) distribution has a shape parameter of 2 leading to an increasing failure rate. So when the shape parameter is 1/2, failures will tend to occur earlier in the interval [0,*τ*] and when the shape parameter is 2 failures will tend to be later.

From Table C1, we see that the proposed design is relatively more cautious when toxicities occur earlier in the interval and more aggressive when toxicities occur later. This will generally lead to more toxicities if onset is later, increasing the average number of toxicities by 0.75 across the 10 scenarios. When the optimal dose is a higher dose level, this aggressiveness leads to a higher probability of choosing the correct dose. When the optimal dose is lower, aggressive dose escalation leads to decreased accuracy in selecting the optimal dose level.
